# OSR1 is a novel epigenetic silenced tumor suppressor regulating invasion and proliferation in renal cell carcinoma

**DOI:** 10.18632/oncotarget.15611

**Published:** 2017-02-22

**Authors:** Yixiang Zhang, Yeqing Yuan, Pei Liang, Xiaojing Guo, Ying Ying, Xing-sheng Shu, Michael Gao, Yingduan Cheng

**Affiliations:** ^1^ Department of Urology, The Second Affiliated Hospital of Jinan University, Shenzhen People's Hospital, Shenzhen, Guangdong, China; ^2^ Department of Urology, David Geffen School of Medicine, University of California Los Angeles, Los Angeles, California USA; ^3^ Department of Pathology, The Second Affiliated Hospital of Jinan University, Shenzhen People's Hospital, Shenzhen, Guangdong, China; ^4^ Department of Physiology, School of Basic Medical Sciences, Shenzhen University Health Sciences Center, Shenzhen, China; ^5^ Institute of Molecular Medicine, Health Science Center, Shenzhen University, Shenzhen, China; ^6^ Twigbiotechnology, Shenzhen, Guangdong, China

**Keywords:** OSR1, methylation, invasion, proliferation, RCC

## Abstract

Renal cell carcinoma (RCC) is one of the most malignant tumors in human. Here, we found that odd-skipped related transcription factor 1 (*OSR1*) was downregulated in 769-P and 786-O cells due to promoter CpG methylation. *OSR1* expression could be restored by pharmacological demethylation treatment in silenced cell lines. Knockdown of *OSR1 in two normal expressed* cell lines- A498 and ACHN promoted cell invasion and cellular proliferation. RNA-Sequencing analysis showed that expression profile of genes involved in multiple cancer-related pathways was changed when *OSR1* was downregulated. By quantitative real-time PCR, we confirmed that depletion of *OSR1* repressed the expression of several tumor suppresor genes involved in p53 pathway, such as *p53*, *p21*, *p27*, *p57* and *RB* in A498 and ACHN. Moreover, knockdown of *OSR1* suppressed the transcriptional activity of p53. Of note, OSR1 depletion also led to increased expression of a few oncogenic genes. We further evaluated the clinical significance of OSR1 in primary human RCC specimens by immunohistochemical staining and found that OSR1 expression was downregulated in primary RCC and negatively correlated with histological grade. Thus, our data indicate that *OSR1* is a novel tumor suppressor gene in RCC. Downregulation of OSR1 might represent a potentially prognostic marker and therapeutic target for RCC.

## INTRODUCTION

Renal cell carcinoma (RCC) is one of the most malignant tumors, which caused more than 140,000 deaths per year [1]. In 2013, more than 350,000 people were diagnosed with RCC worldwide. Smoking tobacco, hypertension and obesity are considered as risk factors for RCC [1]. Despite the development of therapeutic regimens [2], the prognosis of patients with RCC is still poor, mainly due to delayed diagnosis and a relatively high incidence of metastasis. Thus, there is an urgent need for identification of novel diagnosis and therapeutic targets for RCC. However, the molecular mechanism underlying the tumorigenesis of RCC remains elusive.

It is well known that abnormal genetic and epigenetic pattern will lead to tumorigenesis [3]. Currently, it is well accepted that epigenetic alterations even precede genetic changes during tumorigenesis [3]. An increasing number of epigenetic silenced tumor suppressor genes (TSGs) were identified in multiple cancers [4–8], which exert antitumor effects but silenced by promoter methylation in tumor specific manner.

OSR1 gene, located on human chromosome 2p24.1, contains three C2H2 zinc finger domains. OSR1 is reported to be involved in embryonic heart and urogenital formation. It also plays key roles in the development of the metanephric kidney and regulates formation and differentiation of kidney precursor cells. Furthermore, OSR1 is considered as a negative feedback regulator of nodal-induced endoderm development [9–12]. Of note, recent studies have shown tumor specific silencing of *OSR1* by promoter methylation in gastric and lung cancer [13, 14]. Whereas overexpression of *OSR1* significantly inhibited cell growth, arrested cell cycle, and induced apoptosis in the gastric cancer cell lines AGS, MKN28, and MGC803, knockdown of *OSR1* led to enhancement of cell proliferation and inhibition of apoptosis in the normal gastric epithelial cell line GES1[13], indicating that OSR1 is a functional tumor suppressor in gastric cancer.

In this study, we found that *OSR1* expression was frequently silenced in some of the RCC cells, and the expression silencing could be restored by 5-Aza-2′-deoxycytidine (DEC) treatment. Its downregulation was caused by promoter methylation as validated by quantitative methylation-specific PCR (qMSP). Knockdown of *OSR1 in normal expressed cancer cell lines elevated invasion ability and cellular proliferation. RNA-Sequencing of RCC cell lines following OSR1* depletion has identified hundreds of potential target genes of OSR1, which are involved in DNA replication, cell cycle, mismatch repair, p53 and Wnt pathway. A few of downregulated TSGs (*p53*, *p21*, *p27*, *p57* and *RB*) and upregulated oncogenes (*MYC*, *FRA1*, *MET*, *HMGA1* and *PIK3CA*) were further confirmed by real-time PCR. In addition, knockdown of *OSR1* repressed the transcriptional activity of p53. We further evaluated the clinical significance of OSR1 in primary human RCC specimens by immunohistochemical staining and found that OSR1 expression was downregulated in primary RCC and negatively correlated with histological grade. Thus, our data indicate that OSR1 functions as a novel TSG in RCC but is frequently epigenetically silenced in this cancer. Downregulation of OSR1 might represent a potentially prognostic marker and therapeutic target for RCC.

## RESULTS

### Expression profile of *OSR1 in RCC cells*

**Figure 1:**
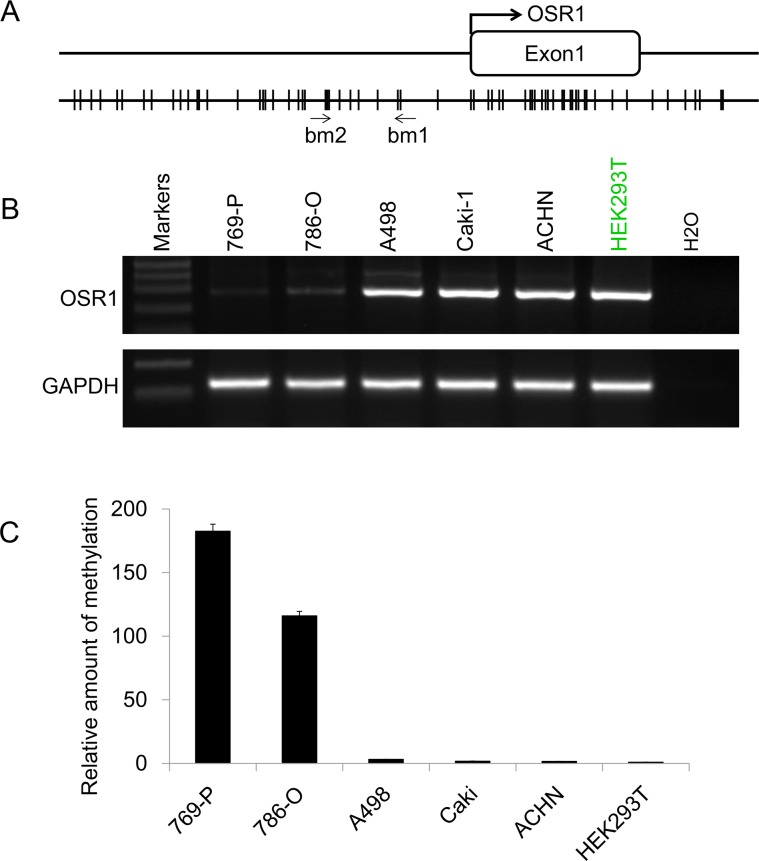
**A.** Schematic structure of *OSR1* promoter CGI. The transcription start site is indicated by a curved arrow. qMSP primers are indicated. Bm1 and bm2 designate primers designed according to the sequence of the bottom strain. **B.** The expression profile of *OSR1 in a series of RCC cell lines. GAPDH* was used as an internal control. **C.** qMSP results of OSR1 promoter in RCC cell lines.

Promoter sequence analysis of the *OSR1* gene revealed a typical CpG island spanning the proximal promoter and exon 1 regions (Figure 1A). We then checked the expression profile of *OSR1 in* five RCC cell lines and one immortalized human renal epithelial cell line HEK293T by semi-quantitative RT-PCR. We found that *OSR1* was expressed in A498, Caki-1, ACHN and HEK293T, but downregulated in 769-P and 786-O (Figure 1B). This tumor specific silenced pattern suggested that *OSR1* was potential silenced by promoter methylation in a tumor specific manner.

### Downregulation of *OSR1* was caused by promoter methylation

To determine whether methylation of *OSR1* results in its downregulation in specific RCC cell lines, the methylation status of *OSR1* promoter was examined by qMSP with primers *OSR1*bm1 and *OSR*1bm2 (Figure 1A). *ACTB* was used as internal control to monitor the DNA quantity and quality. We found that promoter of *OSR1* was methylated in 769-P and 786-O, where expression of *OSR1* was downregulated, but not in cell lines of ACHN, A498, Caki-1 and HEK293T, where *OSR1* was normal expressed (Figure 1C). Our data suggested that promoter methylation of *OSR1* led to its downregulation in RCC.

### Pharmacological demethylation restored *OSR1* expression in RCC cell lines

**Figure 2:**
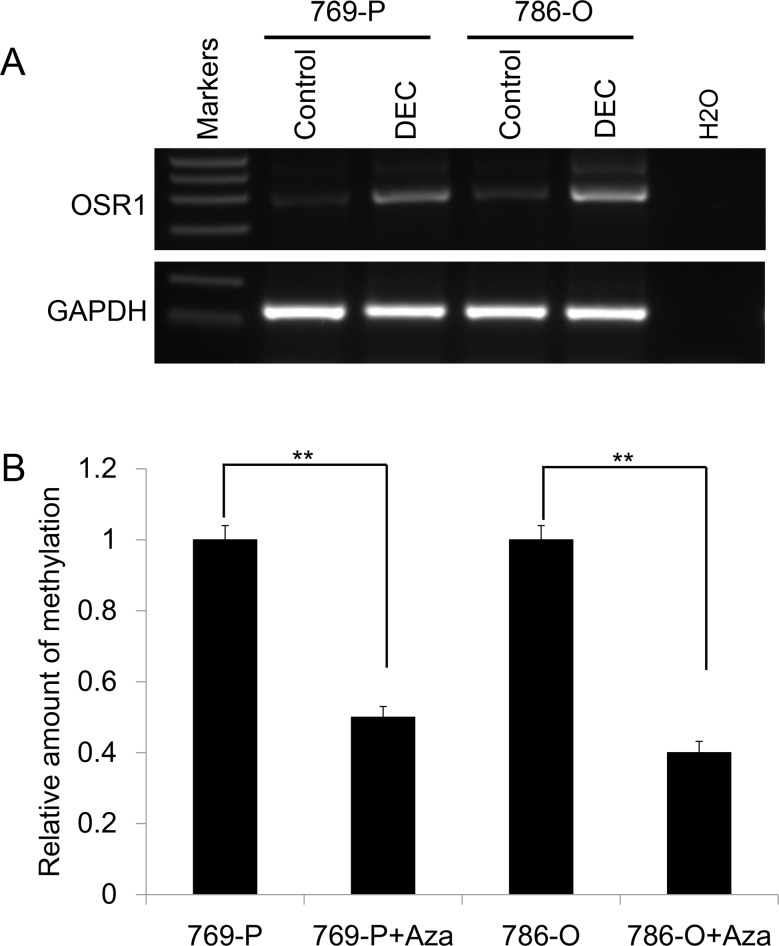
**A.** Pharmacological demethylation with DEC restored the expression of *OSR1 in silenced cells.* **B.** *qMSP results of OSR1* promoter in pharmacological demethylated cells and untreated cells.

To further validate our hypothesis that downregulation of *OSR1* was directly mediated by promoter methylation, 769-P and 786-O cells with methylated and downregulated *OSR1* were treated with DNA methytransferase inhibitor DEC. Pharmacological demethylation treatment with DEC resulted in the upregulation of *OSR1* expression (Figure 2A) accompanied by a decrease in the methylated alleles of *OSR1* (Figure 2B) in 769-P and 786-O cells. These results indicated that downregulation of *OSR1* was directly caused by promoter methylation in RCC cells.

### Loss of *OSR1* promoted cell invasion in RCC

**Figure 3:**
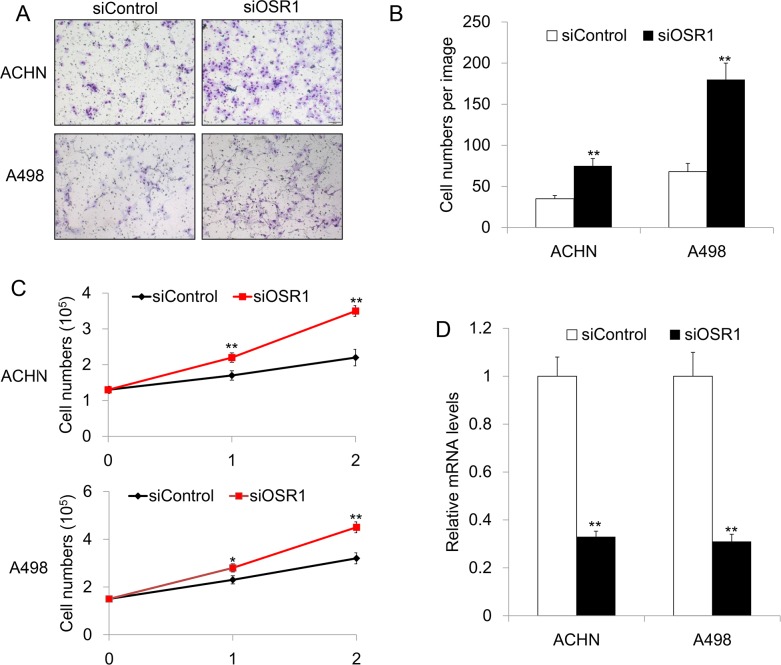
**A.** Representative invasion image of *OSR1 in siControl-transfected or siOSR1-transfected ACHN and A498 cells.* **B.** *Quantitative analysis of invasive cell numbers in siControl-transfected or siOSR1-transfected ACHN and A498 cells*. ^**^, *P*<0.01. **C.** Growth curve of ACHN and A498 cells without or with *OSR1* silencing, respectively. ^**^, *P*<0.01. **D.** Knockdown efficacy of *OSR1 in ACHN an A498 cells*. ^**^, *P*<0.01.

Previous study showed that *OSR1* is a functional tumor suppressor in gastric cancer [13]. The expression profile of *OSR1 in RCC indicated that OSR1 might also have tumor suppressor function in renal cancer*. In order to investigate the role of *OSR1 in* RCC, siRNA knockdown of *OSR1* was performed in RCC cell lines of A498 and ACHN that show normal *OSR1* expression. We examed the role of *OSR1 in renal cancer cell invasion by transwell invasion assays. The number of siOSR1-transfected A498 or ACHN cells observed on the filter was significantly increased compared with the number of siControl-transfected cells (P*<0.01). Our data revealed that knockdown of *OSR1 in A498 and ACHN increased* RCC cell invasive ability *in vitro* (Figure 3A&3B), suggesting that *OSR1* is a negative regulator of cell invasion in RCC.

### Loss of *OSR1* enhanced cellular proliferation in RCC

We further test proliferation rate in *OSR1* knockdown cells. Firstly, we seed the cells at appropriate density in six-well plate. After 16 hours, cells were transfected with siControl or siOSR1, respectively. Cell numbers were counted at 0 h, 24 h and 48 h after transfection. Interestingly, we found that loss of *OSR1* lead to higher proliferation rate in both ACHN and A498 cells (Figure 3C&3D), indicating that *OSR1* could inhibit cell proliferation in RCC cells.

### *OSR1* regulated multiple genes expression

**Figure 4:**
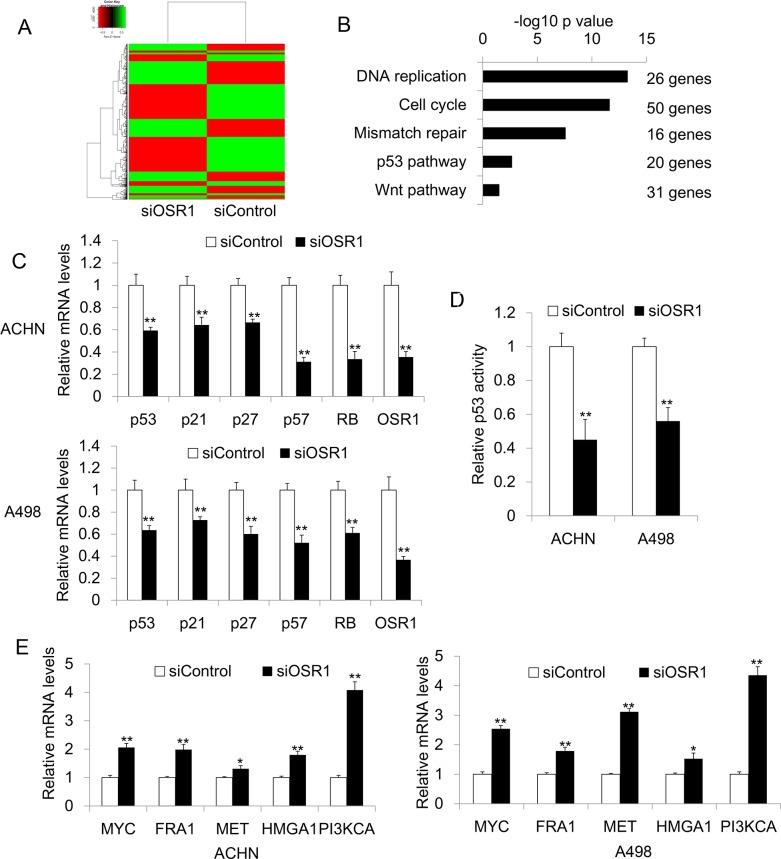
**A.** Heatmap for RNA sequencing results from *OSR1* knockdown ACHN cells and control ACHN cells. **B.** Go analysis for RNA sequencing results. **C.** Confirmation of downregulated genes in ACHN and A498 cells by quantitative real-time PCR. ^**^, *P*<0.01. **D.** P53 luciferase assay in siControl-transfected or siOSR1-transfected ACHN and A498 cells. ^**^, *P*<0.01. **E.** Validation of upregulated genes by real-time PCR in ACHN an A498 cells. *, *P*<0.05; ^**^, p<0.01.

Our functional study suggested that *OSR1* is a functional tumor suppressor in RCC. To explore the underlying mechanism by which *OSR1* exerts tumor suppressive function in RCC, we performed RNA-Sequencing analysis to identify genes that were differentially expressed in *OSR1* knockdown ACHN cells and control ACHN cells. Genes with 2 fold changes were considered as significant (Figure 4A). Firstly, we analyzed the candidate genes by Go analysis. We found that most of the downstream genes are involved in DNA replication, cell cycle, mismatch repair, p53 and Wnt pathway (Figure 4B). The involvement of those cancer related pathway indicated that *OSR1* has functional role in tumorigenesis.

We further confirmed the expression of potential *OSR1* target gene by quantitative real-time PCR. In both ACHN and A498 cells, knockdown of *OSR1* inhibited the tumor suppressor genes, including *p53*, *p21*, *p27*, *p57* and *RB* gene expression (Figure 4C). Moreover, *OSR1* knockdown clearly suppressed *p53* promoter activity in ACHN and A498 cells (Figure 4D). In addition, we found that loss of *OSR1* increased the mRNA levels of several oncogenes including *MYC*, *FRA1*, *MET*, *HMGA1*, and *PIK3CA* (Figure 4E). Our data suggested that *OSR1* acted as a TSG through regulating multiple TSGs and oncogene expression.

### OSR1 was downregulated in primary RCC and correlated with histological grade

**Figure 5:**
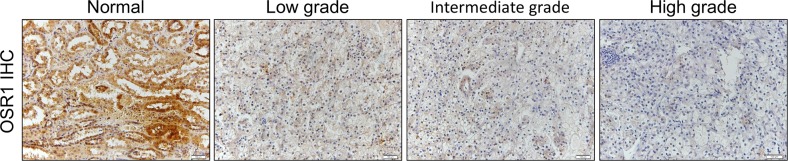
**A.** Representative IHC image of OSR1 in primary RCC samples. OSR1 was downregulated in patient samples and negatively correlated with histological grade of primary RCC.

Our study suggested that OSR1 is a silenced tumor suppressor in specific RCC cell lines due to promoter methylation. We further investigate the clinical significance of OSR1 in primary human RCC specimens by immunohistochemical staining. We found that OSR1 was downregulated in 82.7% (62/75) primary RCC tissues (Table 1, Figure 5). Its expression was significantly lower in primary RCC tissues compared to that in normal tissues (*P*<0.0001, Table 1). Of note, OSR1 expression was negatively correlated with histological grade (*P*=0.002). However, no correlation was found between OSR1 expression and age, gender, and clinical stage. Our data suggested that downregulation of OSR1 might represent a potentially prognostic marker for RCC.

**Table 1:** Relationship between Clinicopathological Variables and OSR1 Expression Level in RCC Patients

Classification	Number	Low expression, n(%)	High expression, n(%)	*P*
Tissues				
Normal	75	20(26.7)	55(73.3)	<0.0001*
RCC	75	62(82.7)	13(17.3)	
Age (year)				
<60	29	23(79.3)	6(20.7)	0.542
≥60	46	39(84.8)	7(15.2)	
Gender				
male	50	40(80.0)	10(20.0)	0.524
female	25	22(88.0)	3(12.0)	
Clinical stage				
I~II	52	44(84.6)	8(15.4)	0.503
III~IV	23	18(78.3)	5(21.7)	
Histologic grade				
poorly differentiated	28	26 (92.8)	2(7.1)	0.002*
moderate differentiated	22	20 (90.9)	2(9.1)	
well differentiated	25	16(64)	9(36)	

Low expression including no(−) and weak(+) staining, high expression including moderate (++) and strong (+++) staining.

## DISCUSSION

In this study, we identified *OSR1* as a novel TSG in RCC. We found that *OSR1* was downregulated by promoter methylation in RCC cells. Inhibition of *OSR1* promoted cell invasion and proliferation. Expression profile of genes involved in multiple cancer-related pathways was changed when *OSR1* was downregulated. A few of representative downregulated TSGs (*p53, p21, p27, p57* and *Rb*) and upregulated oncogenes (*Myc, Fra1, MET, HMGA1, STAT2, PIK3CA* and *L1CAM*) were further confirmed by real-time PCR. We also found that *OSR1* was downregulated in primary RCC and correlated with histological grade. Thus, our present study indicated that *OSR1* is a novel TSG in RCC but is frequently silenced by promoter methylation in this cancer. Downregulation of *OSR1* might represent a potentially prognostic marker and therapeutic target for RCC.

Development of RCC from a normal cell is a complex and multi-step process with multiple oncogenes, TSGs and signaling transduction pathways involved in this process [1–3, 15–20]. Increasing numbers of promoter methylated TSGs identified in RCC [21–23] contribute to elucidating the molecular mechanisms of RCC tumorigenesis. Here, we identified *OSR1* as a novel TSG in RCC. *OSR1* contains three C2H2 zinc finger domain. Previous studies suggested that *OSR1* was involved in embryonic heart and urogenital formation, development of the metanephric kidney, negative feedback regulator of nodal-induced endoderm development [9–12]. *OSR1* expression is also regulated by Runx2 and Ikzf1, which are known as master-gene of osteogenesis and hematopoiesis, respectively [24]. But the function of *OSR1 in cancer is largely unknown. Previous study of OSR1 in gastric cancer suggested that OSR1* is a functional tumor suppressor in gastric cancer. It is frequently silenced by promoter methylation in gastric cell line and primary tumor samples [13]. The present study demonstrated for the first time that *OSR1* is a novel TSG in RCC, which is downregulated by promoter methylation. Remarkly, *OSR1* depletion promoted renal cancer cell invasion and proliferation at least partially through p53 pathway and other important cellular regulators.

The p53 pathway can regulate the basic cellular activity such as proliferation, apoptosis, cell cycle and cellular senescence [25]. Upon a stress signal, activated p53 will bind to p53-responsive DNA sequence elements in the genome. It increases p21 for cell cycle arrest which results in proliferation inhibition. P27 is involved in cell cycle progression and acts as a tumor suppressor to control both tissue expansion and cell proliferation [26]. p57(KIP2) regulates several hallmarks of cancer, including cell invasion, metastasis, apoptosis, and angiogenesis [27]. Tumor suppressor RB contributes to a diversity of cellular functions, including cell proliferation, differentiation, cell death, and genome stability [28]. Interestingly, genes involved in p53 pathway, such as p21, p27, p57, and RB, were significantly downregulated in *OSR1* knockdown RCC cells. Besides, we further confirmed the effect of *OSR1* on p53 pathway by luciferase assay. We found that knockdown of *OSR1* significantly downregulates p53 activity in ACHN and A498 cell lines, which further confirmed our finding. In consistent with our finding, previous study of OSR1 in gastric cancer also found that *OSR1* upregulates p53 in gastric cancer. The role and the regulation of p53 were also reported and summarized in many studies [23, 29–33]. TSGs Mir-22 and ATS/TMS1 could regulate p53 activity in RCC cells [30, 32]. The p53 also regulates several TSGs in RCC [23, 34]. All these suggested that OSR1 functions as a critical TSG in RCC in part through regulation of p53 signaling pathway.

Besides its effect on tumor suppressor genes, we also found that repression of *OSR1* led to increased expression of a few of oncogenes. We confirmed the upregulation of several oncogenes by real-time PCR, including *MYC*, *FRA1*, *MET*, *HMGA1* and *PIK3CA*. *MYC* is correlated with cell growth, proliferation and apoptosis [35]. FRA1 is a component of AP-1 transcription factor complex, which could promote the cell ability of invasion and migration [36]. MET has been implicated in a variety of cellular processes, including cell proliferation, survival, migration, motility and invasion [37]. HMGA1 upregulates cellular proliferation and invasion in multiple cancers [38]. PIK3CA has been shown to be important for tumor cell survival, adhesion, motility and proliferation [39]. The real-time PCR results could explain how *OSR1* downregulates invasion and proliferation in RCC. Our future work will focus on how *OSR1* regulates those genes' expression.

We further investigate the clinical significance of OSR1 in primary human RCC specimens and found that OSR1 was downregulated in primary RCC tissues. Importantly, *OSR1* expression was negatively correlated with histological grade, indicating a potential role of OSR1 as a prognostic marker for RCC.

In summary, we found that *OSR1* was downregulated in RCC cells by promoter methylation. *OSR1* can function as a tumor suppressor via inhibition of invasion and proliferation in RCC cells, possibly via upregulating tumor suppressor genes and downregulating oncogenes. Downregulation of OSR1 was observed in primary RCC and its downregulation was correlated with histological grade, making it a potentially prognostic marker and therapeutic target for RCC.

## MATERIALS AND METHODS

### Cell culture and transfection

A series of RCC cell lines (769-P, 786-O, A498, Caki-1, and ACHN) and an immortalized human embryonic kidney cell line - HEK293T were used for this study. Dulbecco's Modified Eagle Medium (DMEM) supplemented with 10% fetal bovine serum and 1% penicillin/streptomycin was used for cell culture. Cells were cultured in DMEM in a humidified chamber maintained at 37°C and 5% CO_2_. OSR1-short interfering RNA (siOSR1) and control siRNA (siControl) were purchased from Santa Cruz Biotechnology (Dallas, TX, USA). Transfection was carried out according to the manufacturer's instruction using RNAiMAX transfection reagent (Invitrogen, Eugene, OR, USA).

### Pharmacological demethylation with DEC

The method of DEC treatment was described before [4, 6]. In brief, ACHN cells or A498 cells (1 × 10^5^/mL) were allowed to grow overnight in 10 cm cell culture dishes. The cell culture medium was replaced with fresh medium containing 50 μmol/L DEC for every 24 h, for three consecutive days. Then ACHN or A498 cells were harvested for RNA extraction.

### RNA extraction, semi-quantitative reverse transcription PCR (RT-PCR) and real-time PCR

RNA was extracted with Trizol reagent according to manufacturer's protocol (Invitrogen, Eugene, OR, USA). The cDNA was synthesized using Random hexamers and SuperScript-III (Invitrogen, Eugene, OR, USA). Semi-quantitative RT-PCR was performed for 32 cycles with *AccuPrime*™ DNA polymerases, according to manufacturer's protocol (Invitrogen, Eugene, OR, USA). All primers used are listed in Supplementary Table 1. Quantitative real-time PCR was carried out with the Applied Biosystems 7300 real-time systems using real-time PCR Master Mix (SYBR Green). Each experiment was conducted in triplicate in three independent experiments.

### DNA bisulfite treatment and quantitative methylation-specific PCR (qMSP)

The bisulfite treatment was carried out with the EpiTect Bisulfite kit (QIAGEN) by following the manufacturer's instructions. In brief, around 2 ug DNA was used for each reaction and mixed with 85 μL bisulfite mix and 35 μL DNA protect buffer. Bisulfite conversion was performed on a thermocycler followed the manufacturer's instructions. After that, the bisulfite-treated DNA was recovered by EpiTect spin column and used for qMSP. The qMSP was performed with SYBR Green master mix. To correct for differences in both quality and quantity between samples, ACTB was used as an internal control.

### Growth curve

ACHN or A498 cells were seeded in six-well plates at an appropriate density. After 16 hours, cells were transfected with siControl or siOSR1 through RNAiMax. The cell numbers were counted at 0 h, 24 h, and 48 h after transfection. Each experiment was conducted in triplicate in three independent experiments.

### Invasion assay

Cell invasion assay was performed using 24-well culture plates (Millipore, Billerica, MA) with inserts of 8-μm pore membranes pre-coated with Matrigel (BD Bioscience, San Jose, CA). Briefly, ACHN or A498 cells were transfected with siControl or siOSR1 through RNAiMax at an appropriate density in six-well plates. Twenty-four hours after transfection, cells were trypsinized and transferred to the upper Matrigel chamber in 100 μL of serum-free medium supplementing with 1 × 10^5^ cells. The lower chamber was supplemented with medium containing 10% FBS. The invaded cells were stained with 0.1% crystal violet according to manufacturer's protocol (Fisher scientific, Atlanta, GA, USA) after 48 h. Cell numbers per microscopic image field were counted to compare the invasion ability between siControl and siOSR1. Each experiment was conducted in triplicate in three independent experiments.

### RNA sequencing

RNA was purified with an RNeasy Mini kit (QIAGEN). The RNA-Sequencing library preparation was performed according to the manual of manufacturers (KAPA biosystems). Sequencing reactions were performed with the Illumina HiSeq platform. RNA-seq reads were mapped to the human genome (hg19) using Burrows-Wheeler Aligner (bwa) [40]. We then marked the duplicate reads using picard [41]. The HTseq tool [42] was used to calculate the reads count for each gene. Finally, we used the Reads Per Kilobase per Million mapped reads (rpkm) command in edgeR [43] package to calculate the rpkm of each gene.

### Luciferase assay

To exam the effect of OSR1 on p53 pathway, ACHN or A498 was transfected with p53-luc (Stratagene) and pRL-TK by lipofectamine 2000 (Invitrogen). After 6 hours, medium was changed and cells were transfected with siControl or siOSR1 with RNAiMAX (Invitrogen). After 48 hours, cells were harvested and the activity of p53 was analyzed by Promega dual luciferase reporter assay system. All the experiments were performed in triplicates in three independent experiments.

### Patient samples and immunohistochemistry (IHC)

Primary tumor tissues and adjacent normal kidney tissues from 75 different cases of RCC patients were collected in Shenzhen People's Hospital with patients' permission. IHC was performed on 4-μm sections of formalin-fixed, paraffin-embedded human RCC tissues. Sections were deparaffinized, rehydrated and subjected to heat induced antigen retrieval. After incubation with blocking solution, sections were incubated with anti-OSR1 antibody (Abcam) for 1 h, biotinylated secondary antibody for 30 min, and then with streptavidin horseradish peroxidase for another 10 min. Sections were developed with 3,3′-diaminobenzidine chromogen and further stained with hematoxylin. An H-score was assigned to each tissue based on the product of staining intensity ((−), nostaining; (+), weak; (++), moderate; and (+++), strong) and percentage of stained cells (0-0%, 1-1% to 30%, 2-31% to 70%, and 3-71% to 100%). Chi-squared and Fisher's exact test were performed to analyze the association between OSR1 expression and clinicopathological characteristics.

### Statistical analysis

Data are presented as mean ± standard deviation. Statistical assessments were carried out using Student's *t* test. *P* < 0.05 was considered statistically significant.

## SUPPLEMENTARY TABLE


