# Supplementary material for: OSR1 is a novel epigenetic silenced tumor suppressor regulating invasion and proliferation in renal cell carcinoma
Source: Oncotarget. 2017 Feb 22;8(18):30008–18. doi: 10.18632/oncotarget.15611 (PMC5444721; doi:10.18632/oncotarget.15611)
Supplement: Supplementary file 1 [file oncotarget-08-30008-s001.pdf]

OSR1 is a novel epigenetic silenced tumor suppressor regulating invasion and proliferation in renal cell carcinoma

SUPPLEMENTARY TABLE

Supplementary Table 1: Primer list

Application	Primer name	Sequence (5'→3')	Size (bp)
RT-PCR or Realtime PCR	OSR1F	CGTTCTCAGCTGCTCCTGGTT	299bp
	OSR1R	GTTTTGCTGCCCATTTCGGTA	
	GAPDHF	TCATTGACCTCAACTACATG	131bp
	GAPDHR	TCGCTCCTGGAAGATGGTGAT	
	p53F	GTGAGCGCTTCGAGATGTTC	164bp
	P53R	CCCTTCTGTCTTGAACATGAG	
	P21F	AGACCAGCATGACAGATTTC	140bp
	P21R	ACTGAGACTAAGGCAGAAGA	
	P27F	ATAAGGAAGCGACCTGCAAC	145bp
	P27R	ACGTTTGACGTCTTCTGAGG	
	P57F	GGCGATCAAGAAGCTGTCC	64bp
	P57R	CGCTGATCTCTTGCGCTTG	
	RBF	TGTCAGAGAGAGAGCTTGGT	100bp
	RBR	ACAGATTCCCCACAGTTCCT	
	MYCF	GTGCTCCATGAGGAGACAC	209bp
	MYCR	TTGTGCTGATGTGTGGAGAC	
	FRA1F	TACGTCGAAGGCCTTGTGAAC	155bp
	FRA1R	CTCATCTTCCAGTTTGTCAGTC	
	METF	CGCTGACTTCTCCACTGGTT	126bp
	METR	CACTCCCCATTGCTCCTCTG	
	HMGA1F	GCTCCAAGAAGATCCGCATT	148bp
	HMGA1R	GTGGAAGAGTGATGGCTGGG	
	PI3KCAF	GAACCCTTATGTGACAATGTG	103bp
	PI3KCAR	CACGAGGAAGATCAGGAATG	
